# Effect of Tillage and Planting Date on Seasonal Abundance and Diversity of Predacious Ground Beetles in Cotton

**DOI:** 10.1673/031.010.14134

**Published:** 2010-10-11

**Authors:** R. B. Shrestha, M. N. Parajulee

**Affiliations:** Texas AgriLife Research, 1102 East FM 1294, Lubbock, Texas 79403, USA

**Keywords:** Conventional tillage, conservation tillage, diversity, richness, carabids, planting date

## Abstract

A 2-year field study was conducted in the southern High Plains region of Texas to evaluate the effect of tillage system and cotton planting date window on seasonal abundance and activity patterns of predacious ground beetles. The experiment was deployed in a split-plot randomized block design with tillage as the main-plot factor and planting date as the subplot factor. There were two levels for each factor. The two tillage systems were conservation tillage (30% or more of the soil surface is covered with crop residue) and conventional tillage. The two cotton planting date window treatments were early May (normal planting) and early June (late planting). Five prevailing predacious ground beetles, *Cicindela sexguttata* F., *Calosoma scrutator* Drees, *Pasimachus* spp., *Pterostichus* spp., and *Megacephala Carolina* L. (Coleoptera: Carabidae), were monitored using pitfall traps at 2-week intervals from June 2002 to October 2003. The highest total number of ground beetles (6/trap) was observed on 9 July 2003. *Cicindela sexguttata* was the dominant ground dwelling predacious beetle among the five species. A significant difference between the two tillage systems was observed in the abundances of *Pterostichus* spp. and *C. sexguttata*. In 2002. significantly more *Pterostichus* spp. were recorded from conventional plots (0.27/trap) than were recorded from conservation tillage plots (0.05/trap). Significantly more *C. sexguttata* were recorded in 2003 from conservation plots (3.77/trap) than were recorded from conventional tillage plots (1.04/trap). There was a significant interaction between year and tillage treatments. However, there was no significant difference in the abundances of *M. Carolina* and *Pasimachus* spp. between the two tillage practices in either of the two years. *M. Carolina* numbers were significantly higher in late-planted cotton compared with those observed in normal-planted cotton. However, planting date window had no significant influence on the activity patterns of the other species. Ground beetle species abundance, diversity, and species richness were significantly higher in conservation tillage plots. This suggests that field conditions arising from the practice of conservation tillage may support higher predacious ground beetle activity than might be observed under field conditions arising from conventional tillage practices.

## Introduction

Conservation tillage has been defined as a production system in which 30% or more of the soil surface is covered with crop residue (Reeder 2000; Jasa et al. 2000). The practice of conservation tillage has become commonplace with cotton growers in the U.S. cotton belt (Birdsong and Mitchell 2002). In Texas, some form of conservation tillage is used on approximately 25% of cotton hectares. Cotton production under conservation tillage is more profitable than under conventional tillage due to yield advantages and substantial resource savings (Keeling et al. 1989; Parsch et al. 2001; Zenter et al. 2002). The practice of conservation tillage is designed to conserve soil moisture, reduce nitrogen leaching, enhance soil organic matter, and reduce soil erosion (Lascano et al. 1994; Bronson et al. 2001). Conservation tillage may introduce crop management problems such as soil compaction and thus reduced soil aeration, increased or decreased weed pressure, soilwater depletion due to cover crop transpiration, reduced soil temperatures, and increased activity of some insect pests such as cutworms, thrips, and cotton aphids (Bradley 1995; Burmester et al. 1995; Hill 2000; Nayakatawa and Reddy 2000). Conservation tillage practices have resulted in occasional, severe insect pest problems in the southern United States, including infestations of cutworms, cotton aphids, and false chinch bug, *Nysius raphanus* Howard (Leonard and Emfinger 2002).

Predacious ground beetles are generally an important group of natural enemies in many cropping systems. Ground beetles feed on aphids (Bride and Toft 1997), midges and flies (Floate et al. 1990), coleopteran larvae (Barnes et al. 1990; Brust and House 1990), as well as moths and caterpillars (Laub and Luna 1992; Riddick and Mills 1994). While the ecological role of ground beetles has not been fully appreciated nor exploited in biological control programs, their ecological role, specifically in terms of diversity and population dynamics, has been studied in various ecosystems, including arboreal ecosystems (Villa Castillo and Wagner 2002; Fujita et al. 2008), potato (Werling and Gratton 2008; Koval and Guseva 2008), corn (Floate et al. 2007; Lopez et al. 2005), grasslands (Rochefort et al. 2006; Quinn et al. 1991), wheat (Bukejs and Balalaikins 2008; Elliott et al. 2006), vegetables (Eyre et al. 2009), cotton (Torres and Ruberson 2005; Naranjo 2005), and others. The effects of various ecological factors such as temperature, soil cultivation, soil cover, organic fertilizer, and crop rotation on ground beetle communities in different habitats have also been studied.

Because most ground beetles are sensitive to ecological disturbances, including crop management practices (e.g., irrigation, tillage, planting date, pesticide application, harvesting), ground beetles are used as a bioindicator by which the health of the ecosystem is measured (Rainio and Niemela 2003). As such, there is a renewed effort to conserve ground beetle diversity (Rainio and Niemela 2003). Ground beetle population dynamics has been studied in various crops, including corn (Dritschilo and Wanner 1980), potato (Kromp 1990), wheat (Pfiffner and Niggli 1996), cabbage (Hokkanen and Holopainen 1986), and apple (Riddick and Mills 1994). However, no documented information on ground beetle diversity or population dynamics in cotton from the Texas High Plains is available.

Adoption of conservation tillage has changed crop production practices, directly and indirectly impacting cotton agroecosystems. Changes in farming practices affect crop environments and agronomic sustainability. For instance, conservation tillage influences soil properties and microclimate, which consequently affect the dynamics of crop pests and their natural enemies, weed populations, and irrigation scheduling. Ultimately, growth, development, and yields are all affected. The effect of tillage on cotton growth and yield varies with soil type, geographical location, and other management practices. Scientific research describing the influence of tillage and planting date on arthropod natural enemies is scarce, particularly with regard to the population dynamics of predacious ground beetles from the Texas High Plains (Parajulee et al. 2006). The objective of this study was to determine the species composition and seasonal activity patterns of predacious ground beetles and to evaluate the effect of conservation tillage and cotton planting date on the abundance and diversity of predacious ground beetles in Texas High Plains cotton.

## Materials and Methods

A two-year field study (2002–2003) was conducted at the Agricultural Complex for Advanced Research and Extension Systems farm, near Lamesa, Texas. The experiment consisted of two cropping system treatments (tillage and planting date) at two levels each, deployed in a split-plot randomized complete block design and three replications (total 12 experimental units). Tillage was the main plot factor and planting date was the sub-plot factor in a factorial arrangement.

The tillage treatments included conventional and conservation tillage. Planting date treatments included a “normal” planting date recommended for the Texas High Plains (2^nd^ week of May) and a “late” planting date that represented the crop insurance replanting cutoff date for the region (2^nd^ week of June). Conservation tillage included the shredding of the post-harvest cotton stalks, drilling rye seed (62 kg/ha) between the rows of cotton stubble in the winter followed by chemical termination (Roundup Ultra at 1.5 1/ha, www.monsanto.com) of the cover crop one month before cotton planting, and diking furrows (making dikes in every other furrow for rainwater collection and water conservation) once in mid-July. Conventional tillage included shredding the post-harvest cotton stalks, breaking soil with a small spring- tooth implement, bedding, compacting and smoothing the beds, pre-plant furrow diking, weeding (breaking the beds with rod like implement to kill weeds), and in-season furrow diking (three instances). Normal planted plots were planted in cotton on 8 May 2002 and 9 May 2003 whereas the late planting plots were planted on 10 June 2002 and 11 June 2003. Cotton, Paymaster 2326RR (www.deltapine.com), was planted in 1.02 meter rows, in 16.32 meter wide and 30.5 meter long plots, with a plant density of approximately 153,000 plants per ha. The soil texture was Amarillo fine sandy loam. No infurrow application of insecticide for thrips control was made. The cotton seed was not treated with insecticides. The crop was irrigated (35.3 cm in 2002 and 20.8 cm in 2003) by a center pivot system equipped with low energy precision application nozzles and drag socks (Bordovsky et al. 1992). Herbicides were used as needed. Herbicide use included one application of trifluralin (Treflan®, www.dowagro.com) @ 0.6 kg AI/ha in conventional tillage and three applications of pendimethalin (Prowl®, http://www.basf.com) @ 2.24 kg AI/ha and two applications of glyphosate (Roundup Ultra®) @ 0.84 kg AI/ha in conservation tillage. Annually, both tillage systems received 112-38-0 kg N-P-K per ha.

Ground beetles were monitored every 2 weeks from May 2002 to September 2003 using pitfall traps. Traps were made from 710-ml plastic drinking cups submerged in the soil. Two traps were set in the furrows of the middle two randomly selected rows in each of 12 plots, and were used as subsamples for each experimental field unit (a plot). The cups were filled to two-thirds of capacity with a water-detergent solution to aid in holding the beetles after falling into a trap. Dead beetles were collected 48 hours later and were then washed, dried, and identified in the laboratory. Five prevalent ground beetle species, including *Cicindela sexguttata* F., *Calosoma scrutator* Drees, *Pasimachus* spp., *Pterostichus* spp., and *Megacephala Carolina* L. (Coleoptera: Carabidae) were counted and recorded for each trap. Occasionally, pitfall traps caught several non-target insect species such as flies, wasps, moths, and nonpredacious beetles. However, those non-target and non-predacious arthropods were not within the scope of our research objective and thus were discarded while processing the pitfall samples.

For each sample date, species composition data derived from pitfall trap counts were analyzed to estimate the ground beetle's species diversity, species richness, and species evenness. Species diversity was calculated as D = *e^H′^* (Hill 1973, Parajulee et al. 1997), where *H*′ = - Σ(*P_i_.lnP_i_*); *P_i_* = proportional abundance of the *i^th^* species (Shannon and Weaver 1949). Species richness (R) was calculated as the total number of species present in the habitat (Hill 1973, Ludwig and Reynolds 1988). Species evenness, the distribution of species abundances among species, was calculated as E = [(1/λ)-1)]/(e^H′^ 1); H′ and *P_i_* are defined as above (Simpson 1949; Hill 1973; Ludwig and Reynolds 1988). Because pitfall trap samples can only capture the ground crawling adult beetles, this study was specifically limited to ground beetle species. While the species diversity of the entire ecosystem is not represented, ground dwelling beetle species diversity is reflected.

Abundance, diversity, richness, and evenness data were analyzed using analysis of variance (ANOVA), with year, tillage system, and their interaction as sources of variability (PROC MIXED, SAS Institute 2009). Mean separation of treatment effects was performed using least significant difference at α = 0.10 level. The two-year data were combined and analyzed using a single model. The effect of year was analyzed by assigning year as a main plot random source of variation (McIntosh 1983). For any response variable, if year x tillage or year x planting date interaction was significant, data were analyzed for each year separately.

## Results and Discussion

### Species composition

**Figure 1.**
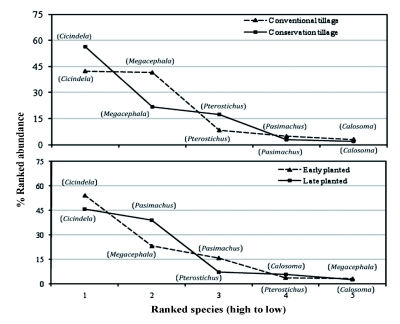
Ranked abundance plot of predacious ground beetle species in a cotton field, Lamesa, Texas (2002–2003). High quality figures are available online.

An analysis of data from 576 pitfall traps (totaling 1,503 beetles) from both years (2002–2003) revealed that *C. sexguttata* was the dominant (50.43%) ground dwelling predacious beetle in the cotton fields, followed by *M. Carolina* (29.87%), *Pasimachus* spp. (12.11%), *Pterostichus* spp. (4.66%), and *C. scrutator* (2.93%). Species composition varied significantly with the tillage system. Two-year combined data analysis showed that the proportion of *C. sexguttata* was significantly higher (65%) in conservation tillage plots (*df* = 1,4; *F* = 8.8, *P* = 0.04) compared with that in conventional (35%) tillage plots. In contrast, *M. Carolina* abundance was significantly higher (68%) in conventional tillage plots (*df* = 1,2; *F* = 2.5, *P* = 0.18) compared with that in conservation (32%) tillage plots in 2003. The rank abundance plot (Figure 1) shows that the ground beetle community in cotton was generally occupied by two species (*C. sexguttata* and *M. Carolina*) while the remaining three ground beetle species collectively contributed less than 20% of the total ground dwelling beetle complex.

Domination of the ground beetle complex by two species may be attributed to habitat disturbances. As agricultural practices intensify, the agroecosystem more closely resembles a monoculture with frequent applications of irrigation, fertilizer, and pesticides. Under these circumstances, niche diversity declines, forcing some species to adapt biologically or behaviorally in order to survive ecological disturbances. Moreover, numerous ecological factors can reduce the ground beetle species diversity in a monoculture. Detailed investigation of various ecological factors arising in monoculture systems is recommended in order to assess the primary reason for changes in insect diversity. Holland and Luff (2000) suggested that arable regions are aptly characterized by a low number of carabid species. It has been suggested that anthropogenic habitat disturbances and interspecific competition may cause scarce carabid species numbers (Memela 1993). Intensive tillage practice generally removes weeds, consequently reducing the number of herbivore prey for ground-dwelling predacious arthropods. The resulting prey scarcity may result in interspecific competition among the predacious beetles. While conventional tillage physically destroys predacious ground dwelling beetles, particularly juveniles, herbicides were used for weed control in conservation tillage plots, killing weeds and thus removing insect food sources, that could result in decreased prey density.

### Seasonal activity

**Figure 2.**
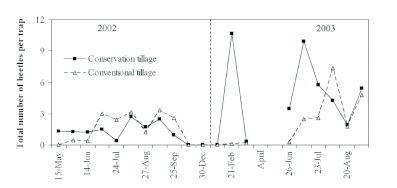
Seasonal abundance patterns of total predacious ground beetles (5 species) in cotton, Lamesa, Texas. High quality figures are available online.

**Figure 3.**
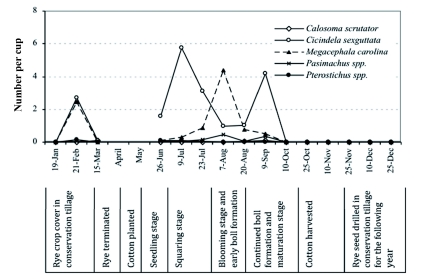
Ground beetle abundance patterns and crop phenology, Lamesa, Texas, 2003. In the months of April and May, sampling was not done due to chemical termination of the cover crop, cultivation or land preparation, and cotton planting. High quality figures are available online.

Ground beetle activity was recorded throughout the year in the experimental plots, except during the period of October–January. Immediately after freezing, ground beetle activity plummeted to a nearly undetectable level where it remained during the following cold months. Ground beetle activity was observed to have resumed in February with the onset of warmer temperatures. Ground beetles were not sampled April and May 2003, due to chemical termination of rye cover and land cultivation for cotton planting. Observed overall ground beetle abundance was lower in 2002 compared with that in 2003 (Figure 2). It is possible that the higher ground beetle numbers in 2003 might have been due to the maintenance of large populations of ground beetles in the conservation tillage plots in 2002. Having been maintained for a year, the habitat might have then provided a source of ground beetles for the new plots in 2003. However, this hypothesis has not yet been tested. Ground beetle activity was low until the beginning of summer (mid-June). It increased with the growth of the cotton crop in the field, and then declined quickly after the onset of colder temperatures in September. In conservation tillage plots, the first ground beetle population peak was observed in February 2003 when the rye cover was flowering. In contrast, ground beetle populations were very low in conventional tillage plots during this period. Ground beetle populations increased as the season progressed, with peak activity detected during July-August (Figures 2, 3). Ground beetle population peaks were more obvious in 2003 versus in 2002 because of the higher overall number of ground beetles in 2003. In 2003, the population peak in conservation tillage was observed one month prior to that of conventional tillage, suggesting more rapid population development in conservation tillage. It is also likely that conservation tillage plots attracted beetles from the neighboring conventional tillage plots, possibly due to greater food source potential, more hiding places, and lower soil temperature in conservation tillage plots during the summer months.

**Figure 4.**
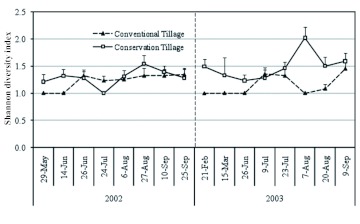
Seasonal dynamics of predacious ground beetle species diversity in conservation and conventional tillage cotton, Lamesa, Texas, 2002–2003. Diversity is shown only for those dates when some ground beetle activity was recorded because the diversity can not be estimated on those dates when no beetle was detected. High quality figures are available online.

**Figure 5.**
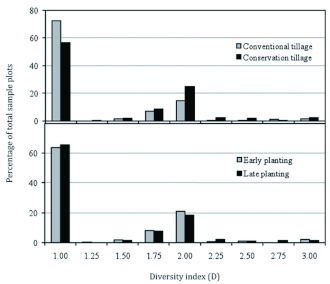
Frequency distribution of Hill's diversity index (D) of predacious ground beetle species in cotton, Lamesa, Texas (2002–2003). High quality figures are available online.

During 2003, observed ground beetle community diversity was low before planting cotton in May (Figure 4). Total ground beetle diversity (both tillage systems combined) slowly increased over time during the cotton growth period, however, this trend could be attributed, primarily, to the faster increase of ground beetles in conservation tillage plots. Ground beetle diversity in conventional tillage plots fluctuated between 1 and 1.5 throughout the study period. Higher beetle diversity was recorded during July–September in conservation tillage plots. Overall species diversity was consistently below 3.0. Most of the samples (approximately 60%) had diversity indices ranging from 0 to 1.0, indicating that there were 0 or 1 species in most of the samples. About 20% of the samples had diversity indices ranging from 1.75 to 2.0 (Table 1, Figure 5). The seasonal dynamics of the ground beetle abundance is not only regulated by habitat disturbances but also by seasonal changes in humidity, temperature, day length (Thiele 1977) and soil moisture (Paarmann 1986). Temperature or humidity extremes influence habitat selection (especially overwintering sites), food availability, and the presence and distribution of competitors (Löver and Sunderland 1996).

**Table 1.**
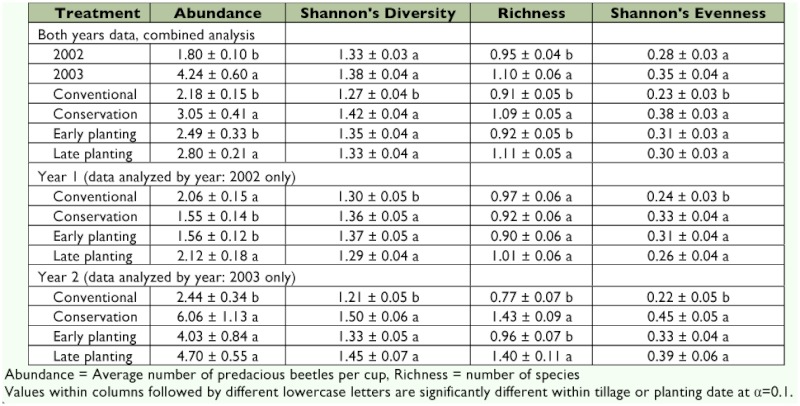
Predacious ground beetle community characteristics (mean ± SE) as influenced by cotton planting date and tillage practice in the Texas High Plains, 2002–2003

### Effect of year

Average predacious beetle abundance was significantly higher (df = 1, 2; *F* = 73.8; *P* = 0.01) in 2003 (4.24 per trap) compared with that in 2002 (1.8 per trap) (Table 1). Average beetle species richness was also significantly higher (df= 1, 2; *F* = 15.8; *P* = 0.05) in 2003 (1.10 species) compared with that in 2002 (0.95 species). However, average species diversity and evenness were similar in both years. The average abundance of *C. sexguttata* was significantly higher (df = 1, 2; *F* = 356; *P* = 0.002) in 2003 (2.4/trap) compared with that in 2002 (0.78/trap) (Table 2). *Pterostichus* spp. and *C. scrutator* populations were significantly higher in 2002 (0.16 and 0.09/trap, respectively) compared with those in 2003 (0.05/trap for both species). More ground beetle species were expected in 2003 versus 2002, speculatively, because the conservation tillage plots that were set up in 2002 provided continuously undisturbed habitat for beetles into the following year. Ground cover left undisturbed throughout 2002 presumably provided a good habitat for overwintering and conditions conducive to early ground beetle propagation, again presumably resulting in higher beetle abundance in 2003. As stated previously, this theory remains untested. Higher precipitation (23.0 cm) was recorded at the study site in 2003 than in 2002 (13.2 cm). *Pterostichus* spp. and *C. scrutator* might have been adversely affected by the higher soil moisture content in 2003 (Paarmann 1986).

### Effect of tillage

Two-year average ground beetle abundance was significantly higher in conservation tillage plots than in conventional plots (Table 1). However, the yearly analysis revealed that while total predacious beetle abundance was higher (df = 1, 2; *F* = 32.9; *P* = 0.03) in conventional tillage (2.06 per trap) in 2002, the abundance was higher (df = 1, 2; *F* = 23.5; *P* = 0.04) in conservation tillage (6.06 per trap) in 2003 (Table 1). Given such an incongruity, it is likely that a better understanding of the effect of conservation tillage on ground beetle dynamics would benefit from a third year of study. In 2003, the observed difference in ground beetle abundance between conservation and conventional tillage was much more pronounced than in 2002.

The two-year combined average ground beetle species diversity was significantly higher (df = 1, 4; *F* = 21.4; *P* = 0.01) in conservation tillage (1.42 Hill's index) than in conventional tillage (1.27 Hill's index) (Table 1). Ground beetle diversity frequency distribution showed that beetle diversity in most plots ranged from 1.75 to 2.0 (Figure 5). Beetle diversity in conservation tillage plots was generally higher than in conventional tillage plots. For most sampling dates, species diversity was higher in conservation tillage plots than in conventional tillage plots (Figure 4). Analysis of combined two-year data revealed that average ground beetle community evenness was also significantly higher (df = 1, 4; *F* = 14.5; *P* = 0.02) in conservation tillage plots (0.38 Shannon's index) than in conventional tillage plots (0.23 Shannon's index) (Table 1). This was discovered to hold true for both years, even when the data were analyzed separately for each year. Similarly, in 2003, average ground beetle species richness was significantly higher (df = 1, 2; *F* = 279.6; *P* = 0.003) in conservation tillage plots (1.43 species) than in conventional tillage plots (0.77 species), whereas the tillage system did not significantly influence the species richness in 2002.

Generally higher carabid diversity (Figure 4) in conservation tillage plots might be indirectly related to higher soil moisture and higher weed/plant diversity. Because the ground cover is presumably capable of supporting more herbivores, the species composition of ground beetles inhabiting the ground cover could be diverse. These observations support the conclusions of Menalled et al. (2007) and Hatten et al. (2007a), who each reported higher carabid diversity in conservation tillage and organic systems compared with that observed in conventional systems.

In a species-specific abundance analysis, *C. scrutator* (df = 1, 4; *F* = 0.6; *P* = 0.46), *M. Carolina* (df = 1, 4; *F* = 2.5; *P* = 0.18), and *Pasimachus* spp. (df = 1, 4; *F* = 2.9; *P* = 0.16) revealed no significant difference in abundances between the two tillage systems (Table 2). Only the abundances of *Pterostichus* spp. (df = 1, 4; *F* = 11.7; *P* = 0.026) and *C. sexguttata* (df = 1, 4; *F* = 8.88; *P* = 0.04) were found to be significantly affected by tillage practice. In 2002, significantly more *Pterostichus* spp. (df = 1, 2; *F* = 86.8; *P* = 0.01) were recorded from conventional plots (0.27/trap) than were recorded from conservation tillage plots (0.05/trap). No significant difference was detected in *Pterostichus* spp. abundance between the two tillage systems in 2003. Significantly more *C. sexguttata* (df = 1, 2; *F* = 10.7; *P* = 0.08) were recorded in 2003 from conservation plots (3.77/trap) than were recorded from conventional tillage plots (1.04/trap). The differences in 2002 were insignificant.

**Table 2.**
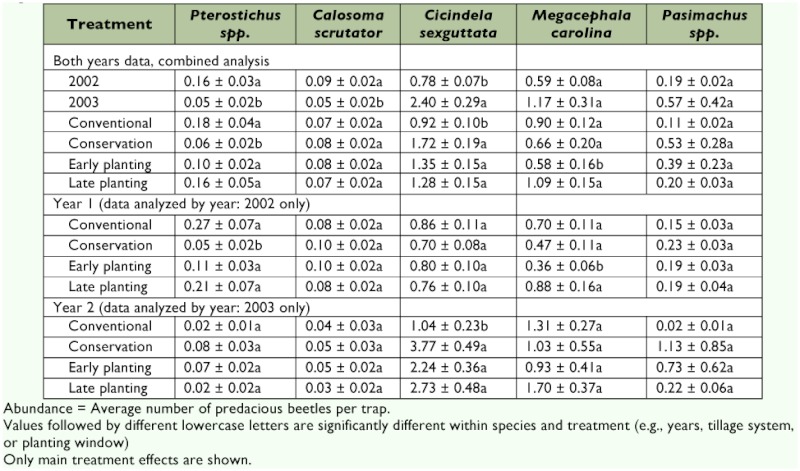
Abundance of ground beetle species (mean ± SE) as affected by cotton planting date and tillage practice in the Texas High Plains, 2002–2003

In contrast to our results, Minarro and Dapena (2003) reported that an herbicide-treated apple orchard under conventional tillage harbored a more diverse carabid community than an orchard mulched with straw. It appears that the effect of tillage practice on carabid diversity is not consistent across cropping systems, possibly due to differences in carabid community structures. Hatten et al. (2007b) also reported the differential effect of tillage on the abundance of three carabid species. The cultivation-induced mortality of late larval and pupal instars has a significant population effect, thus population abundance and diversity of similar carabid assemblages may vary with cropping systems (Purvis and Fadl 2002). While soil cultivation affects a carabid assemblage, a range of results have been reported due to varying local conditions. Theiss and Heimbach (1994) found that carabid larval survival and eclosion rates were adversely affected by high soil moisture, but Davis et al. (2009) reported that the cover crop significantly increased populations of some carabid species in corn. Frank (1997) found higher species richness and activity/density in filter strips than in the crop field. The rye cover planted between the cotton rows in conservation tillage plots in our study might have interacted with the present carabid assemblage in a way similar to that of the filter strips reported by Frank (1997).

### Effect of planting date

In 2003, no significant difference (df = 1, 4; *F* = 1.1; *P* = 0.34) in total predacious beetle abundance between normal planted and lateplanted cotton was observed (Table 1). Total beetle numbers were higher (df = 1, 4; *F* = 32.9; *P* = 0.03) in late planted cotton than in normal planted cotton in 2002, but no significant differences in beetle species diversity (df = 1, 8; *F* = 0.1; *P* = 0.75) and evenness (df = 1, 8; *F* = 0.03; *P* = 0.86) were noted between the two planting dates in either year. Species richness in 2003 was significantly higher (df = 1, 8; *F* = 22.8; *P* = 0.001) in late planted cotton plots versus that observed in normal planted plots, but in 2002, the species richnesses were similar. Analysis of species-specific data revealed that planting date window (early versus late) had no significant effect on the abundances of *C. sexguttata, Pasimachus* spp., *Pterostichus* spp., or *C. scrutator* in either of the two years (Table 2). Of five species and two study years, only *M. Carolina* in 2002 demonstrated a significant response to planting date window. Significantly more *M. Carolina* were collected (df = 1, 4; *F* = 11.8; *P* = 0.03) in 2002 from late planted cotton (0.88 per trap) than from normal planted cotton (0.36 per trap), but the abundances of *M. carolina* were statistically similar between planting date treatments in 2003 (Table 2).

It is possible that more ground beetles were found in late planted cotton due to a longer population development time which was experienced by the ground beetle population in late planted plots prior to planting. Furthermore, early cultivation performed in normal planted plots may have physicially destroyed large numbers of ground beetle juveniles in addition to exposing them to damaging direct sunlight, reducing the overall population in these plots. In addition to destroying juveniles, crop cultivation kills weeds that might serve as hosts, and kills prey insects that might serve as a food source. In this manner, both flora and fauna in the cotton field that might support ground beetle development are disturbed and removed from the system. Soil surface disturbances resulting from tillage hamper predation and expose ground beetles to avian and predatory attack. Ground beetle response to cotton planting date varied between experiment years and among the different species considered. Because the response was unclear and lacked consistency between years, temporal extension of this study and species-specific research are recommended in order to confirm or refute the effect of cotton planting date on ground beetle abundance and diversity.

### Summary

The Texas High Plains region is occupied by the largest contiguously cultivated patch of cotton in the world. The region's cotton agroecosystem differs from cotton agroecosystems in other growing regions due, in part, to its manifestation as a vast continuous monoculture, as well as due to relatively low measured annual precipitation and comparatively low pesticide application utilization in the region. In this study, the seasonal dynamics of carabid ground beetle abundance and diversity in the Texas high plains cotton agroecosystem was examined.

In executing this study, several disadvantages needed to be overcome. Firstly, the study sites were ultimately characterized as harboring minimal ground beetle diversity. Pitfall trap sampling is limited in that its effectiveness in trapping immature ground beetles is questionable. Additionally, as some ground beetles are less active in terms of foraging, it is conceivable that the sampling method was inadequate for capturing the entirety of ground beetle diversity within and extant to the target area. Despite these limitations; however, many ecologists have used pitfall traps in quantifying ground beetle population composition, and the method has been broadly adopted for use in ground beetle sampling, and its effectiveness as a tool in ecological research has been affirmed. Furthermore, only the activity patterns of the adults of prevalent ground beetle species were evaluated. The effects of cover crop and tillage on immature ground beetle growth, development, and activity need to be examined in order to reach a better understanding of factors that induce differences in cotton ground beetle activity.

The precise role of the ground beetle species in cotton insect pest suppression is not well understood, but basically, ground beetles are known to comsume numerous cotton insect pests, aiding in pest population suppression. Generally speaking, a diverse predacious ground beetle population indicates a healthy or undisturbed agroecosystem in which insect pest population suppression is presumed to be greater than that of agroecosystems haboring more homogeneous or uniform ground beetle populations. A more homogeneous ground beetle population, whose composition might be dominated by an abundant, highly effective single carabid predator, is exceedingly effective in suppressing insect pest species, regardless of lower predator diversity. In fact, heterogeneity or diversity within a ground beetle population can, ultimately, either catalyze or antagonize cotton insect pest suppression. However, because information pertaining to interaction between the various species comprising ground beetle populations extant to cotton agroecosystems remains unclear, to generalize that predator diversity is directly proportional to pest population suppression would be a mistake.

A detailed study quantifying the ecological role of the major ground beetle species and seeking to understand their behavioral and biological interactions in cotton agroecosystems would be beneficial to this area of research. Species composition and abundance and diversity seasonal dynamics information generated by this study is essential in modeling the ecological role and function of ground beetles. In addition to seasonal variation, the effects of tillage practice and cotton planting date should be considered as key factors in predicting ground beetle activities in cotton agroecosystems.

Apparent differences in species composition and abundance patterns of ground beetles between conventional and conservation tillage systems might be due to multidimensional effects related to soil cover and tillage operations. During winter months, the rye cover crop typically harbors some arthropod pests (prey for ground beetles) in addition to providing protective shelter and a relatively warm micro-environment for each. Therefore, most of the beetle species began to colonize earlier (before or at the time of cotton planting) in conservation tillage. Thus, it is expected that frequent tillage practices (such as “sandfighting,” or soil cultivation aimed at reducing sand storm damage to cotton plants) in a conventional cropping system might disturb the habitat of adult ground dwelling beetles and juvenile development. For some species, however, conventional tillage favored higher abundance and activity than did conservation tillage. This could be due to prey availability differences and/or other factors affecting behavior and survival. Thus, there is a need for a better understanding of the ecology and behavior of these ground dwelling predators in relation to pest populations, soil moisture, temperature, soil structure, and ground cover availability. A full understanding of the field-level biology, behavior, and in particular, the foraging ecology of the various carabid species is yet to be achieved.
